# A Quantitative Image Cytometry Technique for Time Series or Population Analyses of Signaling Networks

**DOI:** 10.1371/journal.pone.0009955

**Published:** 2010-04-01

**Authors:** Yu-ichi Ozaki, Shinsuke Uda, Takeshi H. Saito, Jaehoon Chung, Hiroyuki Kubota, Shinya Kuroda

**Affiliations:** 1 Department of Biophysics and Biochemistry, Graduate School of Science, University of Tokyo, Bunkyo-ku, Tokyo, Japan; 2 CREST, Japan Science and Technology Agency, University of Tokyo, Bunkyo-ku, Tokyo, Japan; Virginia Tech, United States of America

## Abstract

**Background:**

Modeling of cellular functions on the basis of experimental observation is increasingly common in the field of cellular signaling. However, such modeling requires a large amount of quantitative data of signaling events with high spatio-temporal resolution. A novel technique which allows us to obtain such data is needed for systems biology of cellular signaling.

**Methodology/Principal Findings:**

We developed a fully automatable assay technique, termed quantitative image cytometry (QIC), which integrates a quantitative immunostaining technique and a high precision image-processing algorithm for cell identification. With the aid of an automated sample preparation system, this device can quantify protein expression, phosphorylation and localization with subcellular resolution at one-minute intervals. The signaling activities quantified by the assay system showed good correlation with, as well as comparable reproducibility to, western blot analysis. Taking advantage of the high spatio-temporal resolution, we investigated the signaling dynamics of the ERK pathway in PC12 cells.

**Conclusions/Significance:**

The QIC technique appears as a highly quantitative and versatile technique, which can be a convenient replacement for the most conventional techniques including western blot, flow cytometry and live cell imaging. Thus, the QIC technique can be a powerful tool for investigating the systems biology of cellular signaling.

## Introduction

The mathematical modeling of signaling networks based on quantitative measurements of signaling activities is essential for a systems understanding of signal transduction [1], [2], [3], [4]. For this purpose, large amounts of data with a high numerical precision, and a high spatio-temporal resolution, are desirable. There are a variety of techniques for quantitative measurements of signaling activities. Among them, western blotting, flow cytometry, and live cell imaging are the most commonly used, owing to their specialty (Table 1). However, there is no versatile and high throughput technique for quantitative measurements of signaling activities, including phosphorylation and the localization and expression of signaling molecules, in adherent cells with single cell resolution.

**Table 1:** Comparison between quantitative assays for the signal transduction study.

Characteristic	Quantitative image cytometry	Western blot	Flow cytometry of fixed cells	Live cell imaging
Automated sample preparation	Yes	No	No	Not required
Possible signaling interference	No	No	Detaching adherent cells	Introduction of Fluorescent probes
Resolution of quantification	Subcellular structure	Population of cells	Single cell	Subcellular structure
Sample format	Fixed adherent cells	Lysed cells	Fixed cell suspension	Live adherent cells
Phospho-signal	Yes	Yes	Yes	Limited
Probe	Antibody	Antibody	Antibody	Fluorescent proteins
Temporal analysis	Snapshot	Snapshot	Snapshot	Time-lapse
Signal specificity	Antibody specificity and cellular localization	Antibody specificity and molecular weight	Antibody Specificity	Specificity of Fluorescent probe
Multiplex assay	Yes	No	Yes	Yes

Live cell imaging is a unique technique that provides time-lapse data of signaling activity with high spatio-temporal resolution, which is ideal for investigating the translocation kinetics of signaling molecules such as the nucleo-cytoplasmic shuttling of extracellular signal-regulated kinase (ERK) [5], [6]. However, the application of live cell imaging is limited by the availability of fluorescent probes compared with other techniques that utilize antibodies as probes. In addition, the introduction of exogenous fluorescent probes into target cells sometimes leads to controversial results (see Discussion). By contrast, the western blot is regarded as the most versatile homogeneous assay for quantifying signaling activities, in part due to the availability of specific antibodies. It is also regarded as a convenient and quantitative assay; however, practical automation has so far not been implemented. Simultaneous multiple signaling activities with single cell resolution can be obtained by the use of flow cytometry. In combination with intracellular phospho-protein staining techniques, flow cytometry is intended for measuring signaling activities and has been shown to provide similar qualitative results to western blot analyses [7]. However, since flow cytometry is applicable only to suspended cells, adherent cells have to be detached into a single cell suspension prior to the analysis, which possibly perturbs the signaling activity of interest.

By contrast, image cytometry techniques consist of microscopic imaging techniques and computer aided image analysis that can preferentially analyze adherent cells. Image cytometry techniques can accommodate most staining techniques developed for flow cytometry, and are amenable to automatic sample preparation because each solution-phase reaction can be carried out in place, where cells are fixed on, using a liquid handling system. Another advantage of image cytometry over flow cytometry is that the spatial characteristics of individual cells, such as morphologic characteristics and molecular localizations, are more easily quantified. Taking these features together, image cytometry is usually regarded as a high-content screening (HCS) technique, and is most extensively utilized in fields such as drug discovery [8] and genomic profiling [9] that require high-throughput and high-content screening. Despite the advantages, image cytometry has not been widely applied to the study of signal transduction because image cytometric analysis of signaling activities is considered to be qualitative rather than quantitative, similarly to immunostaining techniques, which are considered to be inferior in uniformity and reproducibility.

The accuracy of image cytometry depends on both sample preparation and imaging, and also largely depends on image processing. The conventional approach of image analysis, which is widely used for the HCS assay to determine signaling activities in individual cells, comprises the initial identification of individual cells by nuclear specific staining; signal intensities are then summed in the nuclear regions plus the cytoplasmic regions, defined by an annulus around each nucleus [10]. This means that the summed signal intensity does not reflect the total intensity in a cell. Therefore, the quantification with this approach is not equivalent to flow cytometric analysis, although the population average would be equivalent to western blot analysis. This approximate algorithm is widely used and, consequently, image cytometry is not directly comparable to the conventional techniques because it is computationally difficult to automatically identify whole cells as compared to individual nuclei, which are relatively easy to identify. In particular, subconfluent adherent cells, such as PC12 cells, are often touching each other, and most cells have rough and obscure cell boundaries. Therefore, it is difficult to identify the cell contour of such adherent cells and to obtain quantitative measurements that are comparable to those from flow cytometric analysis. To overcome such difficulties, a novel image-processing algorithm is required.

**Figure 1:**
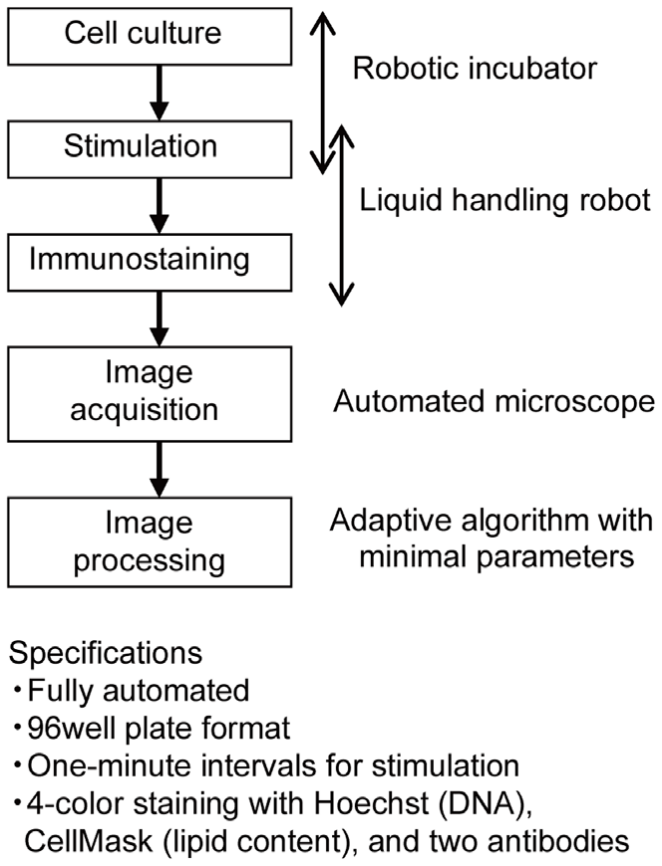
A conceptual diagram of quantitative image cytometry.

Based on the above shortcomings in the available systems, we developed a high-throughput quantitative assay system, termed quantitative image cytometry (QIC), with image cytometry as a convenient replacement for the most conventional techniques (Figure 1). The QIC technique includes the automation of all experimental procedures, as well as a quantitative immunostaining technique and image-processing. We confirmed that the reproducibility of the QIC analysis was comparable to that of western blot analysis, and that the signaling activities measured by each of the techniques correlated very well. Using the QIC technique, we investigated the signaling activities of MAPK/ERK kinase (MEK) and ERK in PC12 cells, and confirmed that nucleo-cytoplasmic shuttling of ERK is largely dependent on its phosphorylation state, which has been reported for exogenous ERK derivatives in previous reports [5], [6]. From the present results, we discuss the utility of this high-throughput and high-content assay for investigating the systems biology of cellular signaling.

## Results

### A quantitative immunostaining technique and image processing algorithm

There is nothing special about quantitative immunostaining; the essential step to perform quantitative and reproducible immunostaining is to keep the staining condition uniform. We utilized 96-well optical-bottomed plates and a liquid handling system to ensure the exact reaction time for all of the wells in each step in the staining procedure. Standard immunofluorescent staining protocols provided by antibody suppliers were adjusted to the equipment and further optimized following a previous study [7], so as to maximize fold changes (treated/untreated) in staining level (see Methods). In this regard, however, we optimized the dilution rate of the primary antibody, instead of optimizing its reaction time. We also included two additional stains, one for the nucleus and the other for the cytoplasm, with specific fluorophores to facilitate the subsequent image analysis. Images of the stained samples were acquired with a wide field fluorescent microscope, and then signaling activities in individual cells were quantified with the following image analysis.

**Figure 2:**
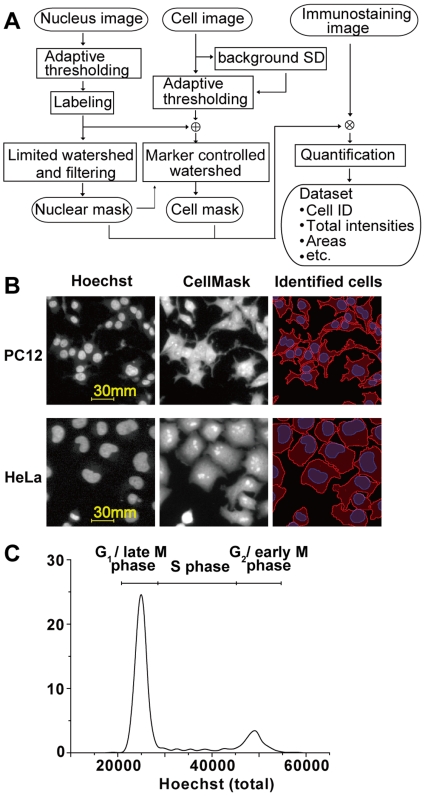
The identification of individual cells by image processing. (A) A block diagram of the image-processing algorithm for cell identification. Circled keywords indicate images, and boxed keywords indicate processes. ⊕ and ⊗ indicate the logical sum and the logical product of images, respectively. (B) PC12 (upper row) and HeLa (lower row) cells were stained with Hoechst (left column) and CellMask Deep Red (center column). Images of stained cells were processed, and the individual cells were identified (right column). The red and blue lines indicate the cell and nuclear contours identified, respectively. (C) DNA histogram of PC12 cells obtained by QIC.

We developed a proprietary image-processing algorithm that precisely identifies the irregular cell contour of PC12 cells (Figure 2A), and implemented it as a plug-in for commercial image processing software (see Methods). Briefly, the algorithm first identifies individual nuclei by labeling bright objects in the nuclear image, in which nuclei are stained with a DNA binding dye. Similarly, entire cell regions are identified by labeling the cell image in which the cells are uniformly stained with a lipid-binding dye. Because the intensity of this nuclear and cell staining was insensitive to cellular signaling activities, cells and nuclei could be stably labeled regardless of the presence or absence of stimulation. Finally, the labeled objects were further divided so that each object had only one nucleus, thus we could obtain the nuclear and cytoplasmic regions in individual cells. The total amount of antigen in a cell and nucleus was quantified as the total fluorescence signal within a corresponding region.

In the labeling step, the segmentation of the bright object and the background is the most crucial step for precise cell contour identification. Conventional automatic segmentation is performed using an appropriate threshold derived from image statistics. However, it is practically difficult to determine a feasible threshold that gives proper segmentation all over the image because the background level of typical microscopic images is not uniform due to light scattering and non-uniform illumination. Instead, we employed an adaptive thresholding approach in which a threshold is determined at each pixel based on some “local” statistics of the neighboring pixels. For instance, a local average at a pixel is calculated as the average intensity of the windowed region surrounding the pixel. For nuclear segmentation, the threshold for each pixel was set at the local average within a window of 41×41 pixels (about 30 µm on a side). For the cell contour segmentation, the threshold for each pixel was calculated as three times the SD above the average of local background intensity, where SD is the standard deviation of the background. In this regard, the local average background intensity was approximated by the average of the presumable background region within a window of 41×41 pixels determined with local average thresholding with the window. However, the SD was substituted with the global background SD because the local background SD became large when the window was close to a bright object. The global background SD was given as the mode of the histogram that consists of the local SD with a window size of 5×5 pixels for all pixels in the image. Note that this local threshold was determined mostly irrespective of the brightness of proximal objects.

The above-mentioned nuclear segmentation technique extracted nuclear regions from the background very well; however, adjacent nuclei were identified as a single object. To separate these clumps into single nuclei, we employed a limited watershed algorithm that recursively separates an object region into two sub-regions only if the lowest watermark by which the object is separated into two regions retains an intensity of more than 85% of that of the parental object. We removed objects for which the total intensity was lower than the appropriate threshold, corresponding to roughly half of the typical Hoechst intensity of cells in G_1_ phase, to eliminate fluorescent particles other than cells as well as apoptotic cell fragments. The remaining objects represent individual cells, and were used as the nuclear mask. Using the marker-controlled watershed algorithm with these nuclei as markers, we obtained the cellular mask by separating the segmented cellular regions so that each region contained a single nucleus. Finally, we obtained the total intensities within the individual nuclear and cellular masks from immunostaining images as signaling activities, as well as corresponding measurements such as the nuclear localization of the antigen (see below).

The algorithm we developed here does not require specific morphological features of cells, and the algorithm can be adapted to various types of adherent cells. Indeed, PC12 cells and HeLa cells could be identified with the same parameters, under the same staining conditions (Figure 2B). Note that because the parameters are set to avoid excessive separation, proximal nuclei are not separated and are recognized as a multi-nucleated cell. Therefore, cells for which the roundness was less than 1.2 were regarded as cells with single nuclei and were used for further study. The crosstalk between different fluorescent spectra and the effect of photo bleaching were negligible (data not shown), and were not corrected. Among the cells used in the analyses of a typical experiment, we found 1.43% cells were ill-identified (see Methods). Although excluding these ill identified cells as well as morphologically irregular cells changed in the analyses slightly, we used whole cells in the following analyses for the sake of fair comparison to other techniques.

The DNA histogram obtained by QIC showed clear separation of cells between G_1_ and G_2_/M phase (Figure 2C). This indicates that the accuracy of the DNA histogram obtained by this method is equivalent to that obtained by flow cytometric analysis. The accuracy of QIC analysis compared with conventional HCS analysis is evaluated in the following sections.

### Validation of QIC technique

**Figure 3:**
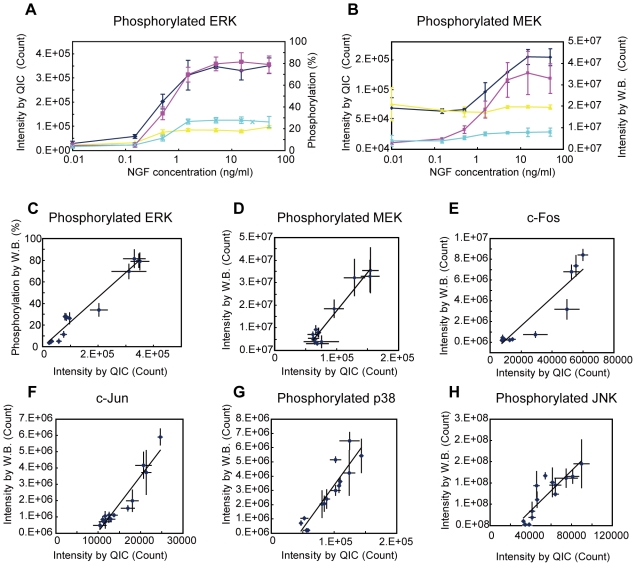
Quantitative image cytometric analyses correlate well with western blot analyses. (A) PC12 cells were treated for 5 minutes or 45 minutes with various concentrations of NGF, and then assayed for ERK phosphorylation using either QIC (blue line for 5 min and yellow line for 45 min) or western blotting (W.B.; magenta line for 5 min and cyan line for 45 min). (B) The same experiments as in (A), but the cells were assayed for MEK phosphorylation. (C, D) The plots shown in (A) and (B) were transformed into scatter plots (C) and (D), respectively, by plotting the measurements of QIC and those of western blotting onto the horizontal and vertical axes, respectively. The lines shown are the regression lines for the points. (E–H) Similar experiments as described above were performed for other antigens as indicated. In (G) and (H), cells were treated with various concentrations of anisomycin for 15 minutes and for 45 minutes. The results represent the average of the normalized measurements (see Methods). The bars indicate the standard deviation of three independent experiments. The average of the total immunofluorescence intensity in individual cells was taken as the measurement for the QIC, and the total luminescent intensity of a specific band (or a set of bands) was taken as the measurement for the western blot analysis.

In order to examine the validity and reproducibility of QIC compared with the western blot, we compared the results of the two analyses with equivalently treated samples of PC12 cells. The concentration-response curves obtained by the two techniques for phosphorylated ERK in response to nerve growth factor (NGF) stimulation for 5 or 45 minutes were quite similar to each other (Figure 3A and Supplementary Figure S1). In contrast to phosphorylated ERK, the concentration-response curves for phosphorylated MEK obtained by the two techniques were different. QIC analysis showed higher basal phosphorylation levels as compared to western blot analysis, although the profiles of the corresponding curves were similar (Figure 3B and Supplementary Figure S2). As a consequence, the QIC analysis for phosphorylated MEK showed smaller fold changes when compared with the western blot analysis. However, the Pearson correlation coefficient between the results of the two analyses for each antibody, which is independent of the baseline drift, was very high (Figure 3C,D and Table 2), indicating that the results of these two analyses are interchangeable by use of a linear function.

**Table 2:** Correlation coefficients and reproducibility.

Antigen	Pearson's Correlation coefficient	Mean CV	Mean CV
		QIC	WB
pERK	0.98	0.14	0.19
pMEK	0.97	0.37	0.23
c-Fos	0.94	0.36	0.23
c-Jun	0.95	0.21	0.24
pp38	0.92	0.24	0.21
pJNK	0.87	0.13	0.22
Mean	0.94	0.24	0.22

The Pearson's correlation coefficients between the QIC and western blot analyses and the reproducibility of each technique calculated from the data set shown in Figure 3 are summarized.

Similarly, the induction of the c-Fos and c-Jun proteins in response to NGF stimulation (Figure 3E,F), and p38 and JNK phosphorylation in response to anisomycin treatment (Figure 3G,H) were analyzed by both techniques. The specificity of the immunostaining was confirmed by the following three points: i) immunostaining images for c-Fos and c-Jun showed inducible nuclear staining [11], those for phosphorylated ERK, p38 and JNK showed inducible diffuse cytosolic staining [12], and those for phosphorylated MEK showed inducible cytoplasmic staining [13], as expected (Supplementary Figure S2, see also Methods); ii) for all antibodies tested by western blot, clear bands were observed at the molecular weights of the antigens with some negligible non-specific bands for some antibodies (Supplementary Figure S1, see also Discussion); iii) for all antibodies tested, the correlation coefficients between the results of the two analyses were very high (Table 2). Significantly, the high correlation with the western blot analysis demonstrates that QIC is a quantitative assay if standards of known amounts of antigen are analyzed by western blot together with the above experiments.

Next, we evaluated the reproducibility of QIC compared with that of the western blot. The reproducibility was calculated as an average of CV values of 14 different measurement points, where each CV value was obtained from 3 independent experiments (Table 2). We exclusively employed a baseline subtraction with the highest possible background for the QIC analysis, which was determined as the smaller value of either the x-intercept of the regression lines in Figure 3C-H or the lowest observed value in all the experiments for each antibody to avoid underestimating the reproducibility of the QIC analysis due to the high background levels. Among the 6 antibodies tested, the QIC analysis showed better reproducibility for phosphorylated ERK, phosphorylated JNK, and c-Jun, and the western blot analysis showed better reproducibility for the rest of the antibodies. However, the differences in the reproducibility were not significant for all antibodies and the overall reproducibility was equivalent. Thus, we concluded that the reproducibility of QIC was comparable to that of the western blot. These results indicate that the QIC technique is a versatile alternative to the western blot technique as long as the appropriate antibodies are available.

### Observational study of cell-to-cell variation in ERK signaling

**Figure 4:**
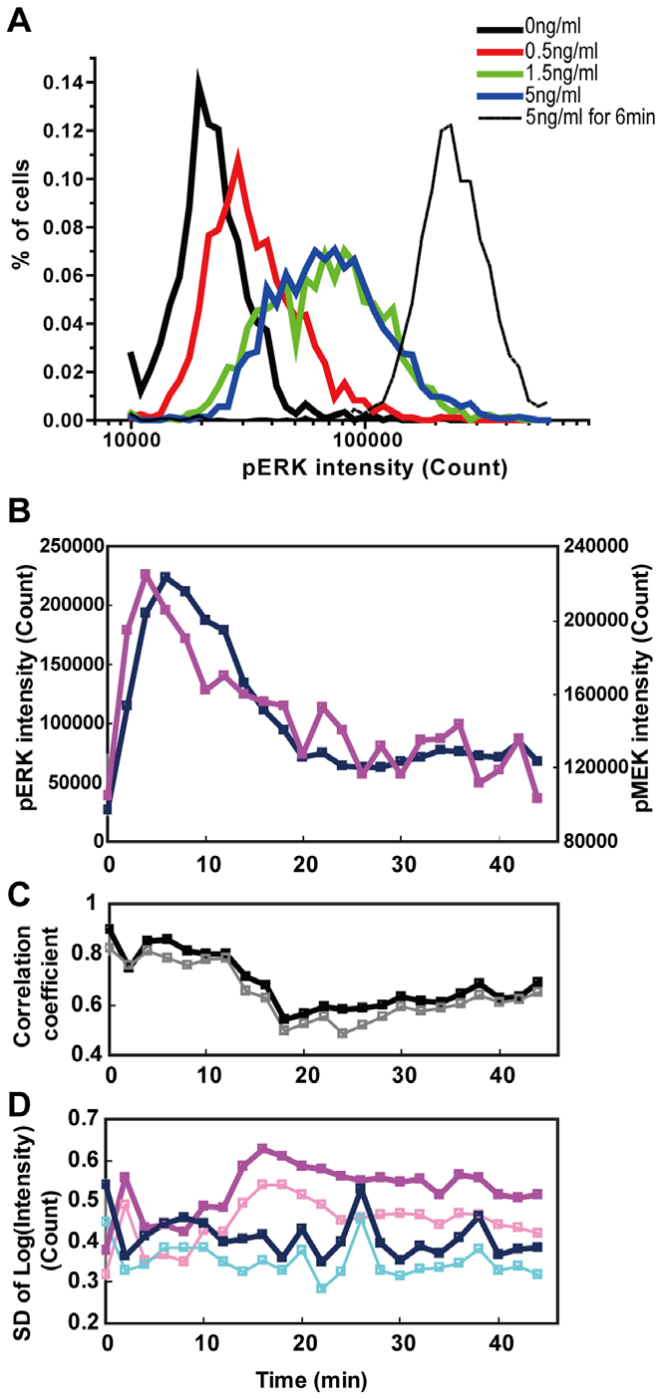
The time series of distribution statistics in ERK signaling. PC12 cells were stimulated with various concentrations of NGF, and then analyzed by QIC with antibodies for phosphorylated MEK and phosphorylated ERK. (A) The single cell distribution of phosphorylated ERK in cells that were stimulated for 6 min (dashed line) or 30 min (solid lines) with the indicated concentrations of NGF. (B-D) Two-minute interval sampling of the distribution statistics in cells stimulated with 5 ng/ml NGF. (B) The averages of phosphorylated MEK (magenta) and phosphorylated ERK (blue). Note that we employed the geometric average for this analysis, which is consistent with the following analyses. (C) The Pearson's correlation coefficient between logarithmically transformed phosphorylated MEK and ERK obtained by the QIC software (black) and the HCS software (gray). (D) The standard deviations of logarithmically transformed phosphorylated MEK and ERK obtained by the QIC software (blue and magenta, respectively) and obtained by the HCS software (cyan and pink, respectively).

One of the advantages of QIC is the ability to quantify cellular signaling activities with single cell resolution. To demonstrate its application to the investigation of intrinsic cell-to-cell variations in signaling activities, we observed the distributions of ERK phosphorylation in response to NGF in PC12 cells (Figure 4A). At 30 minutes after NGF addition, the distributions were always unimodal irrespective of the NGF concentration. Furthermore, because the distributions of ERK phosphorylation and MEK phosphorylation were better approximated by the lognormal distribution than the normal distribution (data not shown), we employed the geometric average to obtain the average of the distribution instead of the arithmetic average, which was used in the above analyses for the comparison with the western blot analysis.

In addition to the cell-to-cell variations, temporal changes in the variation were expected to provide additional information about the signaling network. We observed the distributions of NGF-dependent MEK and ERK phosphorylation simultaneously with sampling intervals of 2 minutes, and calculated the averages, correlation coefficients and standard deviations for MEK and ERK phosphorylation for each time point (Figure 4B-D). The temporal profiles of the averages of both MEK and ERK phosphorylation were similarly transient and subsequently sustained with a slight delay between ERK phosphorylation and MEK phosphorylation. This observation is consistent with the facts that the phosphorylation state of MEK corresponds to its kinase activity, and that phosphorylated MEK directly phosphorylates ERK, leading to ERK activation [14]. By contrast, the time courses of the correlation between MEK and ERK phosphorylation and the standard deviations of both MEK and ERK phosphorylation ran counter to intuition. The correlation coefficient was rather high at the beginning (t = 0) and then gradually decreased for the first 12 minutes after stimulation. It then decreased rapidly until the ERK phosphorylation passed the transient phase. After that, the correlations gradually increased. The standard deviation of the ERK phosphorylation level increased for the first 16 minutes, and then remained at a high level with a gradual decrease. The atypical values we observed at 2 minutes after stimulation were presumably caused by variations in onset of responses in individual cells.

These observations led us to the following hypothetical explanation. In general, phosphorylation level is determined by a balance between specific kinases and phosphatases, and MEK is the only known kinase that directly phosphorylates the activation sites of ERK. Thus, the initial transient phosphorylation of ERK was dominated by the amount of phosphorylated MEK, resulting in a high correlation coefficient. Subsequently, during the transitional phase in ERK phosphorylation from transient to sustained, the dephosphorylation reaction progressively increased its relative contribution to ERK phosphorylation, resulting in a decrease in the correlation and an increase in the standard deviation of the ERK phosphorylation level. This implies that the net activity of phosphatases is maintained independently of the level of ERK activation in response to stimulations (denoted as ‘pathway capacity’ [15]) in individual cells. In addition, the subsequent gradual increase in the correlation and decrease in the standard deviation of the ERK phosphorylation level suggest the existence of ERK phosphorylation-dependent negative feedback regulation for the phosphatase activity. In fact, it has been reported that ERK phosphorylation is involved in at least three negative feedback loops: the induction of ERK-specific phosphatase MKP-1 [16], the inactivation of the ERK pathway regulator SOS [17], and the induction of Sprouty family ERK pathway inhibitor proteins [18], [19]. Although all these mechanisms are likely to be ‘pathway capacity’-dependent, and hence decrease the standard deviation of ERK phosphorylation, only the MKP1 induction can increase the correlation as a consequence of a decrease in the standard deviation of ERK phosphorylation without changing the standard deviation of MEK phosphorylation because MKP1 directly dephosphorylates ERK, but not MEK. Therefore, these collective observations suggest that PC12 cells reduce the cell-to-cell variation in the ‘pathway capacity’ of ERK signaling mainly by the induction of ERK-specific phosphatase. It is worth noting that this kind of analysis can only be achieved through a quantitative time series of simultaneous measurements of signaling activities with single cell resolution. In addition, such analysis is applicable to the study of currently unknown signaling mechanisms.

We analyzed the same image set used in the above experiment with HCS software (Cellomics vHCS software, see Methods) and obtained quite similar results (Figure 4C,D and Supplementary Figure S3). However, the time course of correlation coefficients obtained by the HCS software was shorter in spite of smaller standard deviations of phosphorylated MEK and ERK throughout the observation period. The lessened covariance, as well as smaller standard deviations, was caused by the cross-contamination of signal intensity between neighboring cells as a consequence of the cell identification scheme used in the HCS software, which does not identify individual cell boundaries correctly. This result clearly highlights the importance of precise cell contour identification to the analyses of cell-to-cell variations within a population of adherent cells.

### Translocational analysis with QIC

**Figure 5:**
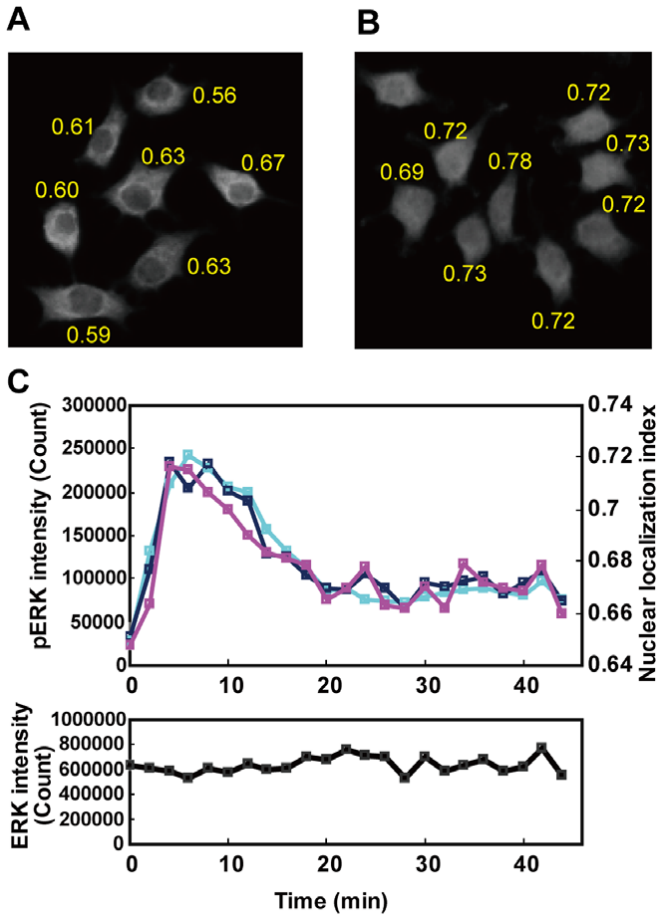
The nuclear translocation and phosphorylation of endogenous ERK. (A, B) PC12 cells were left unstimulated (A) or stimulated (B) with 5 ng/ml NGF for 5 min, and then analyzed by QIC with an anti-ERK antibody. The numbers at nearby cells indicate nuclear localization indices calculated from the cells (see Discussion). (C) PC12 cells were stimulated with 5 ng/ml NGF, and then analyzed by QIC with a combination of antibodies for phosphorylated ERK (cyan line, scale on the left axis) and phosphorylated MEK (not shown), or with a combination of antibodies for phosphorylated ERK (yellow line, scale on left axis) and ERK (magenta line, scale on right axis and black line in lower panel). The upper panel indicates the average of the phosphorylated ERK and the normalized NLI for ERK. The right axis in the upper panel indicates the NLI. The other axes indicate the average of the amount of corresponding antigens in cells. The lower panel indicates the average of the amount of ERK.

Another advantage of QIC is the ability to quantify cellular signaling activities at subcellular resolution. In PC12 cells, it has been reported that ERK translocates to the nucleus in response to NGF stimulation [20], while the total amount of ERK remains constant. To measure the relative nuclear localization of an antigen, we introduced a nuclear localization index (NLI) defined as the cosine value between two vectors of the pixel by pixel fluorescent intensity of either the nuclear staining or antigen staining in a cell (Figure 5A,B and Methods). This index is more solid than the conventional indices [10] because it does not require additional parameters to specify the nuclear and cytoplasmic regions, and is independent from stimulation-dependent fluctuations in the antigen amount and fluctuations in the staining level.

We analyzed the phosphorylation-dependent nuclear translocation of ERK with QIC. PC12 cells were stimulated with NGF, and stained for pan-ERK and/or phosphorylated ERK. The NLI of ERK changed in a similar pattern to the ERK phosphorylation level, whereas the averages of pan-ERK were constant regardless of the phosphorylation state (Figure 5C). This confirmed that the fluorescent intensity of pan-ERK staining actually accounted for the dynamics of the cellular ERK molecules. In addition, the ERK phosphorylation observed in simultaneous staining with pan-ERK was equivalent in both profile and amplitude compared with that without the pan-ERK staining (Figure 5C), confirming that simultaneous staining does not affect the quantification of ERK phosphorylation. The time course of the NLI was in parallel to that of the ERK phosphorylation except for a slight delay seen at the onset. This result was consistent, in principle, with a previous report stating that the time course of nuclear translocation of ERK resembles that of ERK phosphorylation with a slight delay [20]. Moreover, the observation regarding the regulation of ERK translocation completely agreed with a recent report in which time courses of ERK phosphorylation and translocation were simultaneously observed using a fluorescence resonance energy transfer (FRET) probe in EGF-stimulated HeLa cells [5]. Thus, the present evidence demonstrates that QIC analysis allowed us to obtain quantitative data of the translocation of an endogenous antigen at a high temporal resolution.

Next, we compared the results of nuclear translocation analyses obtained with the QIC software with those obtained with the HCS software, which utilizes the Circ/Ring average intensity ratio as a NLI (Supplementary Figure S3, see Methods). We expected that the analyses with the cosine value between nuclear staining and ERK staining would show some advantages over that with the Circ/Ring ratio; however, the analyses of these two NLIs were fairly matched with each other with respect to the time course and single cell distributions of NLIs as well as the antigen amount within the region of quantifications. This is because Ring and Circ regions covered the majority of the cell region while overestimation of the cell region was minimized as a consequence of parameter optimization for the analysis. The present results confirmed that the Circ/Ring ratio is an equally good NLI in comparison to the cosine value as long as analytic parameters are appropriately specified.

## Discussion

Although we optimized our staining protocol and image processing algorithms for analyzing signaling activities in subconfluent cultures of adherent cells, this experimental situation is quite common in the field of cellular signal transduction, where the western blot is commonly utilized. Flow cytometry may also be usable in the same situation; however, we put aside the a direct comparison between QIC and flow cytometry due to some technical disadvantages. Adherent cells have to be detached into a single cell suspension prior to fixation. However, the detaching process severely complicates interval sampling, and the process itself possibly perturbs the labile signaling activity of molecular localization and phosphorylation. In addition, it is difficult to obtain a single cell suspension of some adherent cell lines, including PC12 cells, due to refractory cell-cell adhesion. Noting the absence of these disadvantages for floating cells, QIC and flow cytometry are complementary, rather than competitive, technique to each other. Nevertheless, QIC is potentially comparable to flow cytometry based on evidence that the flow cytometric analysis is comparable to the western blot analysis [7], [21].

We developed a proprietary image-processing algorithm in pursuit of robust and precise identifications of individual cells in subconfluent culture of adherent cells, and demonstrated the advantage of the QIC technique over the HCS technique in the analysis of cell-to-cell variations within a population of adherent cells. There have been a variety of software packages developed for image cytometry both on the commercial and academic sides [22]; however, there are no general purpose software packages designed to address particular situations. Instead, these software packages sometimes provide a programming interface to encourage users to implement specialized algorithms. The current implementation of the algorithm is a plug-in for a commercial image analysis software package, however, it would be easy to port the algorithm onto other image processing platforms, including some open-source platforms such as CellProfiler [23] and CellID [24].

The results of the QIC analysis showed higher basal levels in the signaling activity compared with the western blot analysis for some antibodies tested. This is an inherent disadvantage of immunocytochemical techniques, and represents a trade-off between spatial resolution and signal specificity. However, the higher baseline level does not matter as long as the level is constant across treatments and the measurements are analyzed in a relative manner, as we have shown in Figures 4 and 5. The negative control staining in the absence of the primary antibody demonstrated that the baseline level was substantially dominated by non-specific binding of the primary antibody rather than that of secondary antibody or autofluorescence (data not shown).

The distributions of NGF-dependent ERK phosphorylation measured by QIC were always unimodal, and the mode of the distribution transited gradually according to the NGF concentrations. These results contradict a previous report in which the distribution of ERK phosphorylation measured by flow cytometry was bimodal [3], possibly due to differences in the subtypes of PC12 cells or in experimental conditions such as cell growth conditions.

We introduced an effective NLI for quantifying the nuclear translocation of antigens, that does not require any subsidiary parameters. This index was consistent with the visual evaluation (Figure 5A), robust to fluctuations in the staining level, and also compatible with the HCS analysis with optimized analytic parameters. It is worth mentioning that these features were achieved by the quantification of the amount of antigen through the precise identification of cell contours in individual cells. In addition, the precise identification of individual cells is important for setting up nuclear translocation assays. The staining conditions for a nuclear translocation assay, including the selection of the antibody and its dilution, should be determined so as to maximize the range of the NLI and to minimize changes in the amount of antigen. However, because the localization of the antigen changes depending on the treatment, the amount of antigen is inexactly quantified if one only monitors a subdivision of each cell. Thus, QIC can precisely quantify the nuclear translocation.

It has been reported that the time course of the nuclear translocation of endogenous ERK is qualitatively parallel to that of ERK phosphorylation [20], [25], [26], and it has also been reported that the time course of the nuclear translocation of an exogenous derivative of ERK is quantitatively parallel to its activation state in living HeLa cells expressing the FRET-based probe[5]. The introduction of exogenous probes into living cells inevitably interferes with endogenous signaling activity and thus often requires verification of the functionality and disrupting-effect of the probe. By contrast, our observations are clearly in accord with the presented results, and are free from any interference from exogenous factors.

We have shown that QIC analyses of adherent cells can be used to quantify multiple signaling activities in individual cells as successfully as the flow cytometric analysis of floating cells. QIC is suitable for observing rapidly changing signaling events because of the rapid freezing of cellular processes by direct injection of formaldehyde into the medium immediately after the stimulation period. In addition, with the aid of automation, we were able to observe signaling activities as quantitatively as in a western blot analysis, at every minute after stimulation. These features open a gate to ‘high-content time series analyses’ for investigation of systems biology of cellular signaling networks. In fact, the time series analysis of MEK and ERK phosphorylation in response to NGF in PC12 cells suggested that ERK phosphorylation-dependent ERK phosphatase induction regulates the cell-to-cell variation of the ERK phosphorylation level. In conclusion, QIC can be a powerful and convenient tool for high-content time series analyses.

## Methods

### Antibodies

Mouse anti-phospho-ERK1/2 (Thr 202/Tyr 204) monoclonal antibody (mAb), rabbit anti-phospho-MEK1/2 (Ser217/Ser221) polyclonal antibody (pAb), rabbit anti-ERK1/2 pAb, rabbit anti-phospho-MEK1/2 (Ser221) mAb, rabbit anti-phopho-JNK (Thr183/Tyr185) mAb, and rabbit anti-c-Fos mAb were purchased from Cell Signaling Technology (Beverly, MA). Rabbit anti-ACTIVE p38 was from Promega (Madison, WI). Rabbit anti-c-Jun mAb was from Epitomics (Burlingame, CA). Rabbit anti-ERK1/2 pAb was from Upstate Biotechnology (Lake Placid, NY).

### Cell culture and treatments

PC12 cells (kindly provided by Masato Nakafuku, Cincinnati Children's Hospital Medical Center, Ohio) were cultured at 37°C under 5% CO_2_ in Dulbecco's modified Eagle's medium (DMEM) supplemented with 10% fetal bovine serum and 5% horse serum (Invitrogen, Carlsbad, CA), and stimulated by recombinant mouse beta-NGF (R&D Systems, Minneapolis, MN) or anisomycin (EMD Biosciences, Inc., San Diego, CA) as described. For the QIC assays, cells were seeded at a density of 10^4^ cells per well in 96-well poly-L-lysine-coated glass bottomed plates (Thermo Fisher Scientific, Pittsburgh, PA), and then starved in DMEM containing 25 mM HEPES and 0.1% bovine serum albumin for approximately 17 h before fixation. For the western blot assays, cells were seeded at a density of 8×10^5^ cells per 6 cm plate, and then starved in DMEM for 16 h before treatment. Stimulations for cells seeded in 96-well microplates were performed by replacing the starvation medium with the medium containing the stimulant, using a liquid handling system (Biomek® NX Span-8, Beckman Coulter, Fullerton, CA) with an integrated heater-shaker (Variomag®, Daytona Beach, FL) and robotic incubator (STX-40, Liconic, Mauren, Liechtenstein). Note that all the cells within a plate were fixed simultaneously to prevent the exposure of the cells to formaldehyde vapor during the treatment.

### QIC assays

The cells were fixed by adding 20% formaldehyde directly into the culture medium to obtain a final concentration of 4% formaldehyde. The cells were incubated in fixative for 10 min at 37°C and then washed in ice-cold neutralization buffer (10 mM glycine in PBS), and then in ice-cold PBS. They were incubated with 50% MeOH for 30 min at 4°C, and then washed twice. After this point, the procedures were carried out at room temperature. To permeabilize the cells and to block nonspecific antigenic sites, wells were incubated for 40 min with blocking buffer (0.1% Triton X-100, 10% FBS in PBS). The cells were washed and then incubated for 2 h with primary antibodies diluted in Can Get Signal immunostain Solution A (Toyobo, Osaka, Japan). The cells were washed three times, and then incubated for 1 h with Alexa Fluor 488-conjugated anti-mouse IgG and Alexa Fluor 546-conjugated anti-rabbit IgG (Invitrogen) in the dark, and then washed. After immunostaining, the cells were stained for the nucleus and cytoplasm by incubating with 10 µg/ml Hoechst 33342 (Invitrogen) and 1 µg/ml CellMask Deep Red stain (Invitrogen) solution for 10 min, then washed four times. We used PBS for the dilutions and as the wash buffer unless otherwise specified. The images of the stained cells were acquired using the CellWoRx (Thermo Fisher Scientific) automated microscope with a ×10 objective. For QIC analyses, we acquired two different fields in each well, obtaining 1125±345 (mean ± SD) cells for each well. All liquid handling for the 96-well microplates was performed using a Biomek® NX Span-8 liquid handling system.

### Western blot assays

Stimulated cells were scraped into ice-cold 10% trichloroacetic acid followed by an acetone wash, solubilized with SDS sample buffer, and then subjected to SDS-PAGE. For separation of phosphorylated and non-phosphorylated ERK, low-bis SDS-PAGE (acrylamide:bis  = 144∶1) was used. The resolved proteins were then transferred to nitrocellulose membranes and probed with specific antibodies according to the supplier's instructions. The signal was visualized by chemiluminescence using horseradish peroxidase (HRP)-conjugated secondary antibodies (GE Healthcare, Buckinghamshire, UK) and Immobilon Western Chemiluminescent HRP Substrate (Millipore Corporation, Billerica, MA) with a LAS4000 imager (Fujifilm, Tokyo, Japan).

The intensities of specific bands at the corresponding molecular weights of antigens were quantified by TotalLab TL120 (Nonlinear Dynamics, Newcastle, UK) analysis software. The ratio of phosphorylated ERK2 was calculated from the intensities of bands corresponding to phosphorylated and non-phosphorylated ERK2.

### Image processing and data analysis

The image processing software used in this paper was developed using Visual Basic 2008 (Microsoft, Redmond, WA), and implemented as a plug-in for ImagePro Plus6.1 software (Media Cybernetics, Bethesda, MD).

The roundness of an object was defined 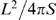, where 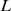 and 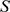 denote the perimeter and area of the object, respectively.

In the analyses shown in Figure 3, we applied an inter-experimental correction to expose the features that are independent of the scaling uncertainty among the experiments. Given 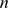 experiments with 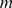 samples for each, we assigned a scaling factor 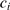 for each 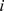-th experiment, and normalized the data set as follows: 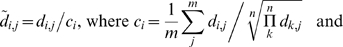




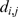
 and 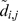 denote the raw and normalized measurement of the 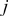-th sample in the 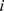-th experiment, respectively. Note that it is often the case in practice that inter-experimental results are compared relative to the internal standard. In this case, all the samples are regarded as internal standards.

The NLI of an antigen in a cell 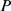 was calculated by the following equation:
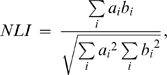



where 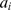
and 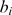 are the intensities of the nuclear and antigen staining, respectively, in a pixel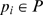. Note that the index ranges from zero to one depending on the subcellular distribution of the antigen, with the corresponding localization from cytoplasmic to nuclear. NLI was slightly affected by cell density, and by staining levels of Hoechst and immunostaining. In the analysis in Figure 5C, we normalized variations of NLIs for ERK staining by the NLIs for the cell staining by the following equation, because the NLI for cell staining was also affected by these parameters.



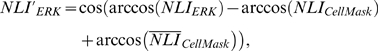



where 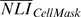 is the average of 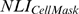 in the well.

Ill-identified cells were specified as those which satisfied the following condition,
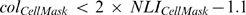



where 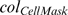 indicates colocalization between Hoechst and CellMask. Note that this type of ill-identification was caused by displacement of the apparent location of nuclei in the cell image from those in the nuclear image, due to the chromatic aberration of the optics system.

Image processing was also performed by commercial HCS software (Cellomics vHCS Analysis Toolbox for cellWoRx version 6.00 with the assay module CellAnalysisToolbox.V2 version 5.0, Thermo Fisher Scientific). The amount of an antigen within a cell was measured as the total amount of correspondent fluorescence intensity in the region of interest (ROI) which was drawn around the nucleus by dilating the nuclear contour. The amount of dilation was set at 16 pixels to maximize the CV value of phosphorylated ERK within a representative image set. The nuclear localization of ERK was measured as the ratio of the average intensity in a circular region (Circ) to that in an annular region (Ring), both of which were drawn around the nuclear contour. The Circ region was drawn by eroding the nuclear contour by 2 pixels. The inner edge of the Ring region was drawn by eroding 1 pixel, and the outer edge was drawn by dilating 4 pixels. These assay parameters were determined so as to maximize fold changes (treated/untreated) of the Circ to Ring average intensity ratio.

## Supporting Information

Figure S1 Gel images of a representative western blot. Representative gel images of the western blot analyzed in Figure 3 in the main text are shown. Arrows indicate the specific bands quantified for the analyses. Note that the contrast of each membrane shown here was adjusted to emphasize non-specific bands.
(2.35 MB TIF)

Figure S2 Images of quantitative immunostaining. Representative immunostaining images analyzed in Figure 3 in the main text are shown. Note that the contrast of each pair of treatments was adjusted so that the background intensity and the relative intensities between the pair of images were preserved.
(7.32 MB TIF)

Figure S3 Image cytometric analyses by HSC software. The same Image sets used in Figure 4 and Figure 5 in the main text were analyzed with HCS software (see Methods in the main text). (A) A representative result of cell identification performed with HSC software. (B) The averages of phosphorylated MEK (magenta) and phosphorylated ERK (blue) analyzed by HCS software. (C) The upper panel shows the average of Circ/Ring average intensity ratio of ERK analyzed with HCS (black lines, scale on the left axis), or the normalized NLI for ERK analyzed with QIC (gray line, scale on the right axis). The lower panel indicates the average of the total amount of ERK in the Circ and Ring region (black lines, scale on the left axis), or the average of the amount of ERK analyzed with QIC (gray line, scale on the right axis). (D) The single cell distribution of the NLIs of ERK in cells that were left untreated (black lines), stimulated by 5 ng/ml NGF for 4 min (red lines) or 30 min (blue lines) analyzed with QIC (left panel) or with HCS (right panel).
(0.97 MB TIF)
