# Supplementary figures and images for: A Quantitative Image Cytometry Technique for Time Series or Population Analyses of Signaling Networks
Source: PLoS One. 2010 Apr 1;5(4):e9955. doi: 10.1371/journal.pone.0009955 (PMC2848603; doi:10.1371/journal.pone.0009955)

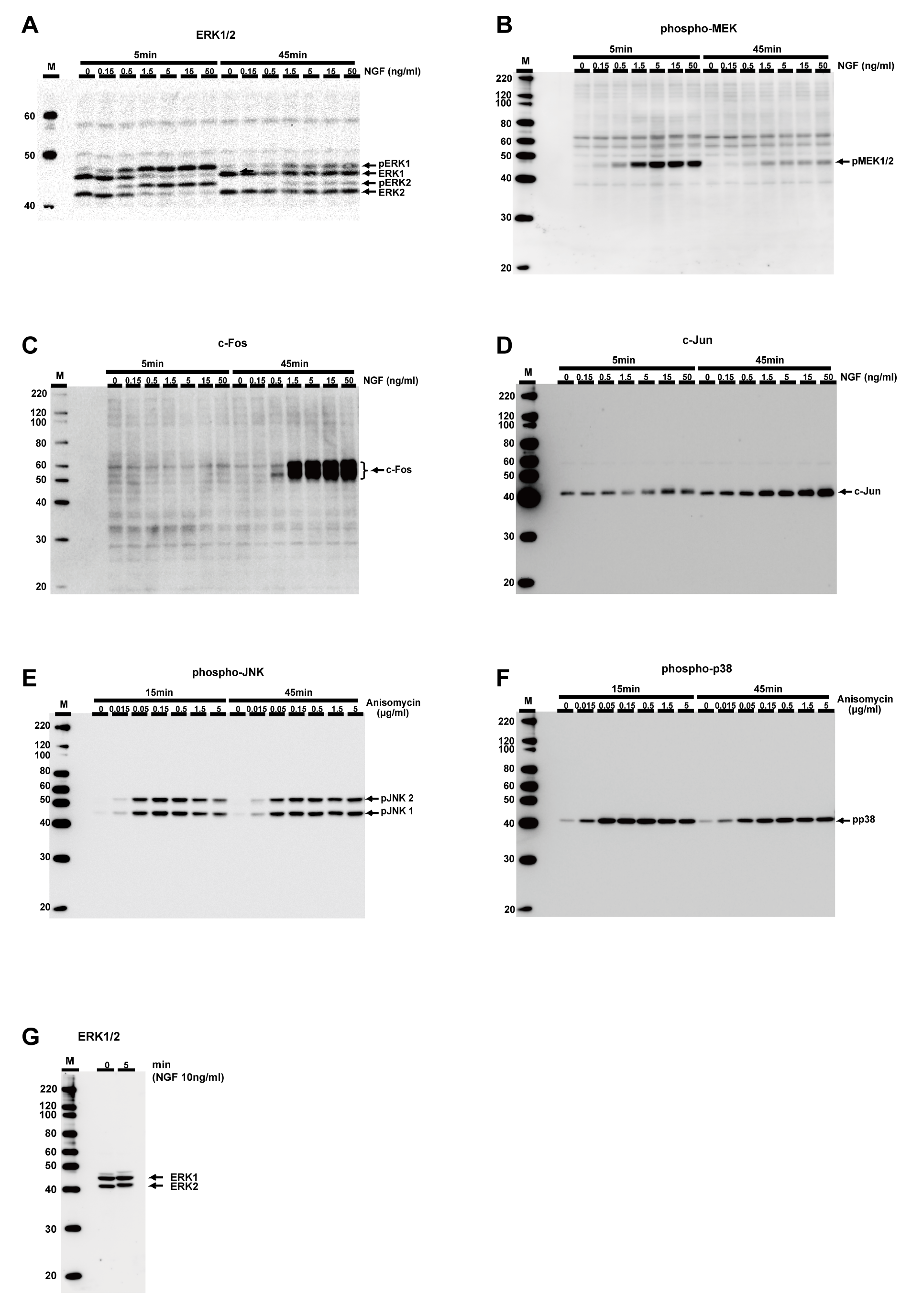

Supplement: Figure S1 — Gel images of a representative western blot. Representative gel images of the western blot analyzed in Figure 3 in the main text are shown. Arrows indicate the specific bands quantified for the analyses. Note that the contrast of each membrane shown here was adjusted to emphasize non-specific bands. (2.35 MB TIF) [file pone.0009955.s001.tif]

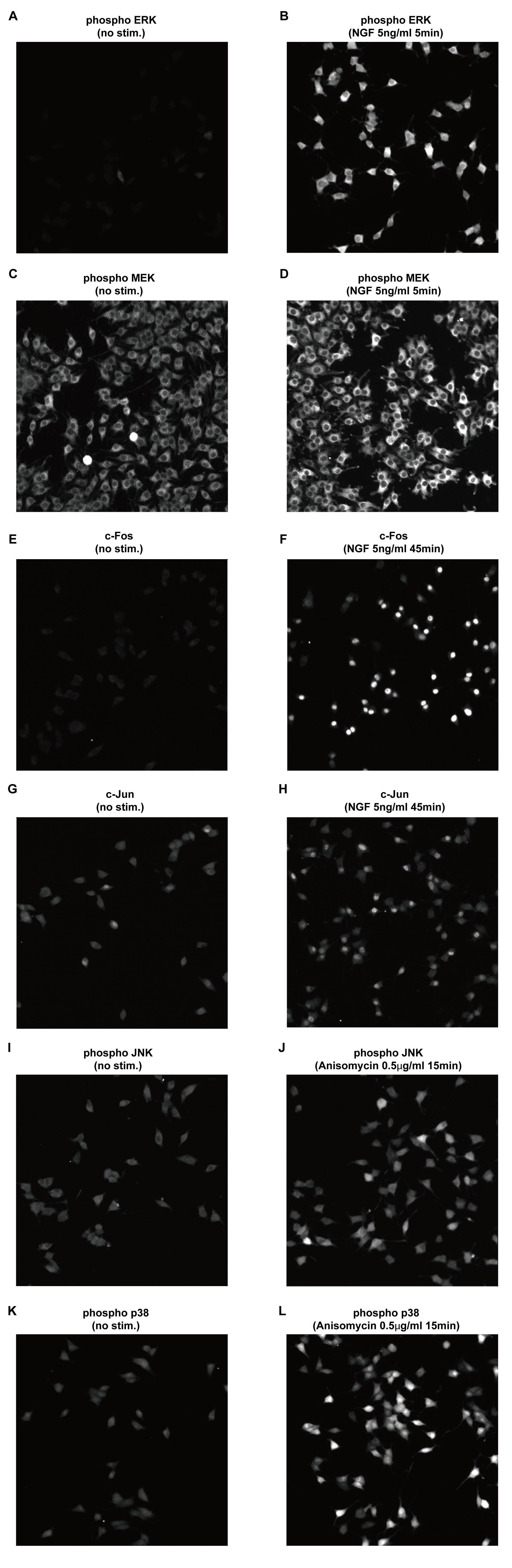

Supplement: Figure S2 — Images of quantitative immunostaining. Representative immunostaining images analyzed in Figure 3 in the main text are shown. Note that the contrast of each pair of treatments was adjusted so that the background intensity and the relative intensities between the pair of images were preserved. (7.32 MB TIF) [file pone.0009955.s002.tif]

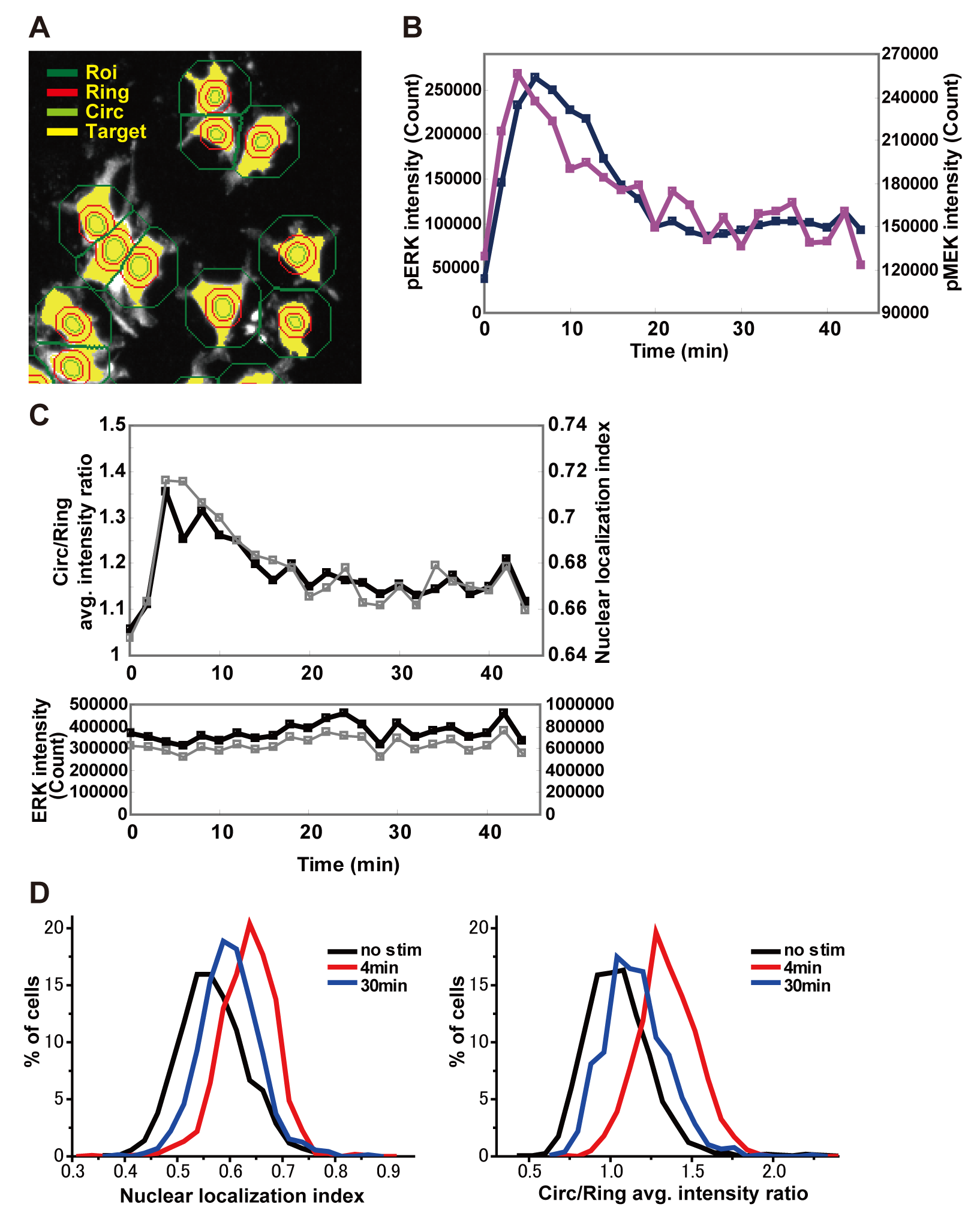

Supplement: Figure S3 — Image cytometric analyses by HSC software. The same Image sets used in Figure 4 and Figure 5 in the main text were analyzed with HCS software (see Methods in the main text). (A) A representative result of cell identification performed with HSC software. (B) The averages of phosphorylated MEK (magenta) and phosphorylated ERK (blue) analyzed by HCS software. (C) The upper panel shows the average of Circ/Ring average intensity ratio of ERK analyzed with HCS (black lines, scale on the left axis), or the normalized NLI for ERK analyzed with QIC (gray line, scale on the right axis). The lower panel indicates the average of the total amount of ERK in the Circ and Ring region (black lines, scale on the left axis), or the average of the amount of ERK analyzed with QIC (gray line, scale on the right axis). (D) The single cell distribution of the NLIs of ERK in cells that were left untreated (black lines), stimulated by 5 ng/ml NGF for 4 min (red lines) or 30 min (blue lines) analyzed with QIC (left panel) or with HCS (right panel). (0.97 MB TIF) [file pone.0009955.s003.tif]
